# Effect of transportation distance and lairage time on selected behaviors and carcass parameters in zebu cattle—a study using the animal focal sampling method

**DOI:** 10.3389/fvets.2024.1385481

**Published:** 2024-05-22

**Authors:** Welligton Conceição da Silva, Amauri Gouveia Júnior, Manoel Soares Damasceno Neto, Luciano Fernandes Sousa, Antônio Vinícius Correa Barbosa, Éder Bruno Rebelo da Silva, Maria Roseane Pereira dos Santos, José de Brito Lourenço-Júnior, Raimundo Nonato Colares Camargo Júnior, André Guimarães Maciel E. Silva

**Affiliations:** ^1^Postgraduate Program in Animal Health and Production in the Amazon (PPGSPAA), Federal Rural University of the Amazon (UFRA), Belém, Pará, Brazil; ^2^Postgraduate Program in Animal Science (PPGCAN), Institute of Veterinary Medicine, Federal University of Para (UFPA), Brazilian Agricultural Research Corporation (EMBRAPA), Federal Rural University of the Amazon (UFRA), Castanhal, Brazil; ^3^Laboratory of Neuroscience and Behavior, Federal University of Pará (UFPA), Belém, Brazil; ^4^Department of Animal Science, Federal University of Tocantins (UFT), Araguaína, Tocantins, Brazil; ^5^Cyberspace Institute, Federal Rural University of the Amazônia (UFRA), Belém, Brazil; ^6^Institute of Engineering and Geosciences, Federal University of Western Pará (UFOPA), Santarém, Brazil

**Keywords:** ethology, bruise, pre-slaughter management, waiting time, cattle

## Abstract

The objective of this study was to evaluate the effect of the transport distance and lairage time on behavioral indicators of zebu beef cattle in the resting pen of a slaughterhouse using the focal animal technique. Eight lots of male zebu cattle, Nelore, aged approximately 4 years old, weighing on average 500 ± 28.5 kg-1, from different municipalities, transported by land, were evaluated. The lots were grouped into two categories: short distance (> 500 km) and long distance (> 500 km). Five focal animals per evaluated lot were used in each cycle of behavior observation, randomly chosen, and visually identified. The animal focal sampling method was used, with instantaneous recording, every 5 min per animal/h. A video camera was used and placed at a fixed point in the corral. After slaughtering, bruises and carcass pH were evaluated. The respiratory rate (RR) was measured in two moments, 30 min after unloading (RR_I) and 1 h before slaughtering (RR_F); behavioral data were divided into four lairage periods (1st, 2nd, 3rd, and 4th) equally and evaluated as repeated measures over time. The lairage time lowered the behaviors of headbutting (NCHBs), pushing (PsH), mounting (MT), and chasing away (CA), (*p* = 0.0001), and these behaviors were more evident in animals transported for short distances in the initial resting times, decreasing as they remained in the resting pen. There was effect of distance on the initial and final pH of the carcass, being greater in the short distance (*p* = 0.047). There was no effect of distance or lairage time on initial or final RR (*p* > 0.05). In addition, animals transported over long distances showed less carcass injuries, which were observed during *post mortem* inspection. It is concluded that animals transported over short distances present a greater number of agonistic behaviors, such as NCHBs, MT, PsH, and CA, predisposing to higher rates of bruising during lairage and more accentuated changes in the final pH of the meat.

## 1 Introduction

The transport of cattle from the property to the slaughterhouse is a pre-slaughter stage that deserves attention, as many factors can affect Animal Welfare (AW) (1), carcass (2), and meat quality (pH, tenderness, and color), and among them, the following stand out: type and capacity of vehicle, distance, travel time, road conditions, space available for animals, driver behavior, and ambient temperature and their associations (3–5).

Another pre-slaughter stage is the lairage time in the slaughterhouse, to provide the animals an environment to recover from physiological efforts due to the loading, transport, and unloading, from the property to the slaughterhouse, which are stressful (6). However, when animals remain in the resting pen for an excessive period of time, fasting and receiving inadequate management, weight loss, dehydration, and fatigue can take place (7–9), which directly reflect on their behavior in the waiting corral.

An important tool for evaluating the AW is the behavior analysis of the animals. When an animal continuously and intensely avoids a situation, information about its degree of well-being is given. Thus, the avoidance reaction, markedly during the presence of a stimulus, may indicate a poor degree of well-being (10).

The behavioral reactions observed in animals can be determined by the way they react to the environment, with animals of other species or the same, as well as humans, which can directly interfere with the change in their posture (11). When animals show normal behavioral characteristics in an unknown environment, it is believed that they have a good degree of AW, for example, the practice of rumination during the resting period is a sign of tranquility (12).

Cattle behavior in the lairage is an indicator of the AW degree, through the identification of the increasing or not of aggressive behaviors (13), which can represent a poor or bad AW degree (12) and reduce the final meat quality.

The behavior evaluation of all the animals in the lot, although relevant, is expensive and, in many cases, prohibitive; more simplified techniques, such as the animal focal sampling method, allow the evaluation of the behavior, without requiring the observation of a large number of animals, being less labor intensive and low cost (14). The objective of this study was to evaluate the effect of transportation distance and lairage time on selected behaviors and carcass parameters in zebu cattle using the focal animal sampling method.

## 2 Materials and methods

### 2.1 Ethical aspects

The experimental procedures were carried out in accordance with Brazilian laws and previously approved by the Animal Ethics Committee (CEUA/UFPA) under protocol number 9307300720.

### 2.2 Location

**Figure 1:**
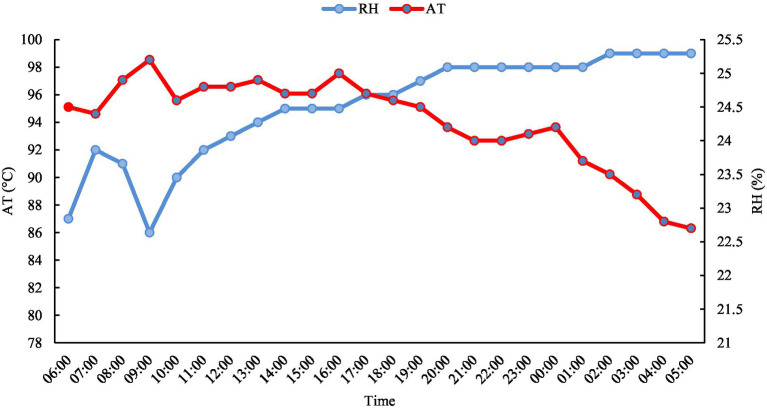
Average air temperature and relative humidity not lowered during the arrival of animals. AT, Air Temperature; RH, Relative Humidity.

The study was carried out in the resting pen of a slaughterhouse, under the Brazilian Federal Inspection Service (Serviço de Inspeção Federal – SIF), in Castanhal, Pará, Brazil. The pen had a rectangular shape with a total area of 112.18 m^2^ in the subdivision, without cover, with metal protection, painted in red. This study was released in February, which is the rainiest period of the year. The floor was paved, non-slip, with water sprinkler system, a drinking fountain, with free access to water. A total of eight farms were used. In this location, the type of climate characterized by Afi (Köppen classification) with average precipitation equivalent to 3.001.3 mm/year, in which the rainiest period is from December to May and the least rainy period is from June to November. In relation, the average annual temperature corresponds to 26.4°C, with an average relative humidity of 84% and annual insolation of 2.338.3 h/year (14). The air temperature and relative humidity during the experiment is shown in Figure 1.

### 2.3 Animals

Eight lots of male zebu cattle (*Bos indicus*), from Nelore breed, acquired from commercial herds that were not crossed with taurine cattle, aged approximately 4 years, uncastrated, weighing 500 ± 28.5 kg, transported by truck, from different properties were studied. The lairage time was not standardized, being equivalent to lot 1–11 h, lot 2–11:45 h, lot 3–12:30 h, lot 4–17:20 h, lot 5–17:25 h, lot 6–17:40 h, lot 7–17:55 h, and lot 8–22:30 h. There was no mixing between lots of cattle evaluated in lairage. The animals remained without food during this period. In addition, for comparison purposes, the lairage time was divided into four periods, each period corresponding to one-fourth of the time spent in the pre-slaughter pen.

### 2.4 Experimental treatments

The most frequent distances performed routinely in the slaughterhouse were selected. The experimental groups were divided into two categories, according to transport distances: short distance (less than 500 km—minimum of 73 km, average of 157.8 ± 59.36 km, and maximum of 303 km), coming from the northeastern microregion of Pará and long distance (greater than 500 km—minimum of 648 km, average of 791.3 ± 95.71 km, and maximum of 863.7 km), coming from the southwestern microregion of Pará, 5 lots short distance, and 3 lots long distance. These distances were estimated taking into account the road route between the headquarters of the municipality where the animals left to the slaughterhouse. The cattle came from farms that breed beef cattle and were not mixed, they came from farms of origin and upon arrival at the slaughterhouse, and they remained in the specific waiting corral for these animals without batch mixing.

The animals were driven in a standard one-story vehicle, a truck (10.60 × 2.40 m), and divided into three compartments: front (2.65 × 2.40 m); intermediate (5.30 × 2.40 m); and back (2.65 × 2.40 m), each compartment held 7 to 8 animals, with an average of 22 animals per truck, transported exclusively by land. The trucks have non-slip floors. Each lot evaluated in the corral consisted of 40 animals, through more than one unloading (truck), always with animals from the same herd (farm). The lots differed in terms of herd and municipality of origin. In terms of transportation, each truck contained 22 animals. The corral had 2.5 m^2^ per animal. All animals were evaluated and slaughtered in the same week.

### 2.5 Focal animal sampling—ethogram

Individuals were observed using the focal-animal sampling technique, with instantaneous recording (15), with observation every 5 min per animal/h (or instantaneous scan sampling—each 5 min 1 observation), with five focal animals every hour, for 25 min (5 min. *5 animals = 25 min) every evaluated hour.

To obtain the images and make the ethogram, a video camera (Hilook THC-B110C-P HD Bullet 2.8 mm; VHD 1120 B G4) was used, located at the back of the corral, and affixed to the edges of the corral held by a 4 m high iron bar.

The behaviors were recorded continuously. Digital video with a high definition (HD) recorder (Seagate® 500 GB) was used in order to store the images. Video data were analyzed using a recording of each animal in Cowlog 3.0.2 Behavior Software (University of Helsinki, Helsinki, Finland) for coding behaviors (16, 17). The evaluated behaviors were pre-defined (Table 1).

**Table 1:** Ethogram of the behavior categories of zebu cattle evaluated during the permanence in the waiting corral.

Behavior	Description
Chasing Away	When an animal comes within 0.5 m of another animal causing the other animal to move away without any physical contact.
Head Butting	Blow Headbutt, quick blow with the head on another animal, in general, without causing the animals to move.
Pushing	When an animal pushes the other with its body, making it move.
Mounting	The act of the animal riding on top of the other animal.
Idleness	Looking carelessly in any direction.
Watching	Staring in a direction to be focused. Generally, the individual remains stationary, although he/she can move the trunk.

Source: Coimbra et al. (18), Agudelo et al. (19) and Silva et al. (20).

To allow the observation of five animals in each evaluation cycle, the methodology proposed by Altmann et al. (15) was adapted, with a new draw every of 60 min evaluation cycle, only with the animals that were fully visible in the camera’s field of view.

At each hour of evaluation, five focal animals were randomly selected in each lot and visually identified, allowing observation through filming. The animals were identified by the phenotypic characteristics expressed by each animal. Thus, according to their characteristics, they were identified.

The cattle belonging to the eight evaluated lots remained at least 11 h, average of 15.75 h and maximum of 22:30 h in the waiting corral. In total, 52.5 h of video were evaluated, considering 25 min per hour, that is, 5 min per animal, according to the adapted methodology (15).

The sessions were divided into hours, in order to make analyses easier. Assessments were made by viewing the footage. To record the behaviors, two trained observers were used alternately in the observations, that is, observer 1 observed animal 1 for 5 min, then observer 2 visualized animal 2, then observer 1 observed animal 3, until 5 animals per hour. Thus, the observers, every 25 min of evaluation, took a 10-min break to rest, and spent a maximum of 4 h per day to evaluate the videos. In this way, the observer’s margin of error (15) is reduced, as a result of the time between sessions, maximizing the accuracy in collecting information.

### 2.6 Respiratory rate (RR)

RR was measured in two moments, 30 min after unloading (RR_I) and 1 h before slaughtering (RR_F), in 15 animals per lot (these being the same animals in RR_I and RR_F), by counting the movements of the animal’s flank, for 15 s, and the value multiplied by four to obtain the number of respiratory movements per minute (21).

### 2.7 *Post mortem* evaluation of carcass injuries

The evaluation of the carcass was carried out immediately after the bovines were slaughtered, with samples of 15 carcasses from each lot, chosen at random, taking into account location, size, age, and degree of bruise. Any area present in the carcass with the accumulation of blood in the tissue, caused by trauma and injury, was considered a bruise (22, 23). Management within the slaughterhouse follows the AW recommendations, with the animals kept calmly until the time of slaughter, avoiding stress levels in the animals. The stunning method consisted of using a penetrating captive dart in the frontal region of the cattle’s head. The batches were slaughtered on different days 2 weeks apart, 4 batches in the first week and 4 batches in the second week.

**Figure 2:**
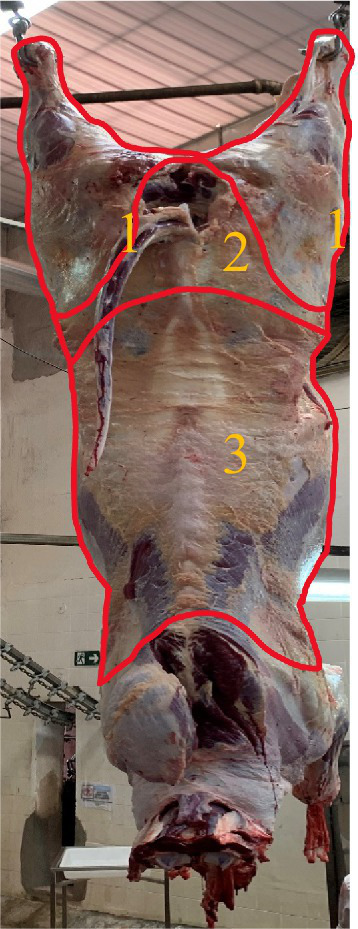
Description of carcass divisions in determining the location and score of bruises in cattle. (1) Thigh region; (2) sacral region; and (3) lumbar region.

In the evaluation of the lesion site, the carcasses were divided into three regions: (1) thigh region; (2) sacral region; and (3) lumbar region (23) (Figure 2).

The size of the bruises was classified on a scale from 1 to 3, according to the lesion diameter: 1—diameter from 1 to 5 cm; 2—diameter from 6 to 10 cm; and 3—greater than 11 cm.

The age of the lesions was estimated based on color. Recent injuries are defined as those resulting from transport and tend to occur at intervals of less than 1 day (22). Lesions were classified as red/bluish or purple corresponding to a time < 1 day and brown to dark purple corresponding to a time > 1 day.

The degree of bruise was classified on a scale from 1 to 2, in which: Grade 1—lesion that affects only the subcutaneous tissue; and Grade 2—injury that affects both subcutaneous tissue and muscle and bone (23).

### 2.8 Meat pH assessment

The pH measurements were performed on 15 carcasses per lot, randomly chosen, using a pH meter (Orion 210A), with a penetration of 5 cm in depth, in the Musculus longissimus dorsi, between the 11th and 13th intercostal space, considering two different times, before and after cooling, 45 min and 24 h after placing in the cold chamber, respectively. The mean temperature after 45 min was 33.5°C ± 2.98 and after 24 h 0.4°C ± 0.32.

### 2.9 Statistical analysis

In the analysis of behavioral data (NCHBs, MT, PsH, and CA), the observation times of each lot (eight) were divided into four periods (the lot lairage time was not standardized in the corral), being them 1st, 2nd, 3rd, and 4th, equally, making it possible to assess the category of behaviors in the initial (1st), intermediates (2nd and 3rd), and final period in the pen (4th). Times were assessed as repeated measures in time.

The design used was completely randomized (CRD) with measurements repeated over time, the main factor being the distance to the slaughterhouse, divided into short (<500 km) and long distances (>500 km) and lairage times (quarters) repeated measurements. Eight lots were assessed, divided into five animals (replica) per hour of evaluation, for each repetition (lot) assessed, divided into two distances considered necessary to mitigate the existing variability, influenced by the number of animals transported by truck and their management by different drivers; length of stay in the pen and its influences on the animal behaviors.

The data were subjected to tests for normality and residuals using W and D statistics (24, 25) and homoscedasticity of treatment variances (26).

In the variables in which the sphericity condition was not accepted, the analysis of mixed models was used, in which all the covariance structures (S) available in the SAS software package (27, 28) were evaluated, which model the dependence of model errors.

The other qualitative variables (NCHBs, MT, PsH, and CA) were organized into contingency tables and evaluated by the *χ*2 index and in quantitative variables (RR_I and RR_F, PH_I and PH_F, and bruise) were evaluated by parametric tests, ANOVA and Tukey’s tests, *post-hoc* parametric tests, after verified their data and residual normality assumptions and equality of variance assumptions. These data mentioned above were analyzed at 5% significance.

## 3 Results

### 3.1 Behavioral data

The focal animal technique allowed the identification of variations in the behavior of the animals, both in terms of distance and lairage time.

There was an interaction between the distance and lairage time factors for all the behavioral variables (*p* = 0.0001) (Table 2). The number of cattle headbutts (NCHBs) for the short distance was higher from unloading until the first half of the lairage time, becoming stable and close to that of the long distance from the second half of the lairage. Lairage time decreased the NCHB, and for short distance, this effect is significant by the second half of lairage time, and for long distance, lairage time reduced NCBSs after the first quarter of time and then stabilized.

**Table 2:** Number of Headbutting (NCHBs), Mounting (MT), Pushing (PsH), and Chasing away (CA) behaviors performed by zebu cattle transported for short and long distances depending on the lairage time (LT) spent in the slaughterhouse pen.

NCHBs (number * 5 min^−1^)									
Distance	Lairage time				Average	*p*			MSE
						Dist	LT	Dist x LT	
	1	2	3	4					
Short^(1)^	2.8860 Aa	2.5000 Aa	1.4060 Ab	0.6480 Ab	1.8600	0.0062	0.0087	0.0255	0.2705
Long^(2)^	1.4500 Ba	0.3360 Bb	0.6660 Aab	0.3420 Aab	0.6985				
Average	2.1680	1.4180	1.0360	0.4950					
MT (number * 5 min^−1^)									
Short^(1)^	0.3000 Aa	0.3320 Aa	0.2000 Aa	0.02000 Ab	0.2130	0.0679	0.0007	0.0122	0.0471
Long^(2)^	0.3340 Aa	0.09400 Bb	0.08560 Bb	0.06600 Ab	0.1235				
Average	0.3170	0.2130	0.1428	0.04300					
PsH (number * 5 min^−1^)									
Short^(1)^	0.1440 Ba	0.1000 Aa	0.08800 Aa	0.01000 Aa	0.08550	0.2637	<0.0001	0.0006	0.0821
Long^(2)^	0.4660 Aa	0.02200 Ab	0.06542 Ab	0.1340 Ab	0.1555				
Average	0.3050	0.06100	0.07671	0.07200					
CA (number * 5 min^−1^)									
Short^(1)^	1.3620 Aa	1.5780 Aa	0.8800 Aab	0.6480 Ab	1.1165	0.0074	<0.0001	0.0019	0.1064
Long^(2)^	1.4500 Aa	0.2000 Bb	0.4980 Ab	0.3000 Ab	0.6120				
Average	1.4060	0.8880	0.6890	0.4740					

Different uppercase letters in the column and lowercase letters in the row point out statistical differences *p* < 0.05, by Tukey’s *post-hoc* test. Dist, distance. LT, lairage time. Headbutt, Blow Headbutt, quick blow with the head on another animal, in general, without causing the animals to move. MSE, Mean Standard Error. *p*, *p*-valor. Mount, act of the animal to mount on the other animal. Push = when an animal pushes the other with its body, making it move. Chase away = when an animal comes within 0.5 m of another animal causing the other animal to move away without any physical contact. Distance: ^(1)^ Short: <500 km (average of 157.8 ± 59.36 km); ^(2)^ Long: >500 km (average of 791.3 ± 95.71 km).

For mounting (MT) behavior, the number was equivalent for short and long distances in the first and last (4th) lairage times, and in the intermediate times (2nd and 3rd), this behavior was higher for short distance. Lairage time reduced MT in animals from short distance only in the last (4th) time, and for long distance, lairage time reduced MT from the 2nd lairage time and then stabilized.

Pushing behavior (PsH) was higher for the long-distance animals only in the first lairage time, while in the other lairage times, the number of pushes was close to that observed previously. Lairage time did not alter PsH in the short-distance animals; in the long-distance animals, this behavior decreased and stabilized from the 2nd quarter of the lairage time onwards.

For the chasing behavior (CA), there was a difference between short and long distance (*p* < 0.0074) only in the 2nd evaluation period, remaining stable in the other periods. For short distances, this behavior was close in the initial periods (1st and 2nd), decreasing and stabilizing in the final two periods (3rd and 4th). Animals transported over long distances showed a higher number of CA in the first few hours in the pen (1st period), with a reduction from the 2nd period onwards and remaining stable in the last periods (3rd and 4th).

### 3.2 Initial and final PH

There was an effect of distance on the initial and final pH of the carcass, with higher initial and final pH for the short distance (*p* = 0.047). The final pH was lower than the initial pH, regardless of the distance (*p* = 0.0001) (Table 3).

**Table 3:** Initial and final carcass pH of zebu cattle, transported for short or long distance, before and after cooling, 45 min, and 24 h, respectively, in the cooling chamber (−2°C to 2°C).

Distance	PH_I	SEM	PH_F	SEM
^(1)^Short	7.047Aa	0.04	6.512Ab	0.71
^(2)^Long	6.909Ba	0.24	5.907Bb	0.24

Different uppercase letters in the column and lowercase letters in the line indicate statistical differences *p* < 0.05 by the chi-square test. PH_I, initial PH (45 min after slaughter, 33.5°C ± 2.98). PH_F, PH_final (24 h after slaughter, 0.4°C ± 0.32). Distance: ^(1)^ Short: <500 km (average of 157.8 ± 59.36 km); ^(2)^ Long: >500 km (average of 791.3 ± 95.71 km). SEM, Standard error of the mean.

### 3.3 Initial and final RR

There was no effect of distance or lairage time on RR (Table 4).

**Table 4:** Initial (RR_I) and final (RR_F) respiratory rates of zebu cattle, transported for short or long distance, in the resting pen of the slaughterhouse.

Distance	RR_I	SEM	RR_F	SEM
^(1)^Short	39.093Aa	0.20	30.613Aa	0.35
^(2)^Long	38.488Aa	0.31	30.222Aa	0.46

Different uppercase letters in the column and lowercase letters in the line indicate statistical differences *p* < 0.05 by the chi-square test. RR_I, initial respiratory rate (30 min after unloading). RR_F, final respiratory rate (1 h before slaughter). Distance: ^(1)^ Short: <500 km (average of 157.8 ± 59.36 km); ^(2)^ Long: >500 km (average of 791.3 ± 95.71 km). SEM, Standard error of the mean.

### 3.4 Bruise

The distribution of bruises by carcass region (thigh, sacral, and lumbar) (*p*-value = 0.1710) was not influenced (*p* < 0.05) by transport distance (Table 5).

**Table 5:** Effect of transport distance on the bruises of zebu cattle’s carcass according to location, size, age, and grade.

Distance	Region			
	Thigh	Sacral	Lumbar	Total
Short^(1)^	16 (21.33%)	35 (46.67%)	24 (32%)	75
Long^(2)^	6 (13.34%)	25 (55.55%)	14 (31.11%)	45
Total	22	60	38	120
Distance	Size			
	1 to 5 cm	6 to 10 cm	>11 cm	Total
Short^(1)^	37 (49.33%) Aa	25 (33.33%)Aba	13 (17.34%)Bb	75
Long^(2)^	19 (42.22%)Ab	10 (22.23%)Ab	16 (35.55%)Ab	45
Total	56	35	29	120
Distance	Age^(3)^			
	< 1 day	>1 day	Total	
Short^(1)^	58 (77.33%)	17 (22.67%)	75	
Long^(2)^	38 (84.44%)	7 (15.56%)	45	
Total	96	24	120	
Distance	Degree^(4)^			
	1	2	Total	
Short^(1)^	29 (38.67%)Aa	46 (61.33%)Aa	75	
Long^(2)^	29 (64.44%)Aa	16 (35.56%)Ba	45	
Total	58	62	120	

Different uppercase letters in the column and lowercase letters in the line indicate statistical differences *p* < 0.05 by the Likelihood Ratio chi-square test. Age^(3)^: < 1 day - Red/bluish or purple; 2 - > (1 day) - Brown to Dark Purple. Degree^(4)^ 1 - Lesion that affects only the subcutaneous tissue; and Grade 2 - Injury that affects both subcutaneous tissue and muscle and bone. Distance: ^(1)^ Short: <500 km (average of 157.8 ± 59.36 km); ^(2)^ Long: >500 km (average of 791.3 ± 95.71 km). Size, Lesion diameter.

There was an effect of distance on the size of the bruises, and animals transported for long distances showed higher percentage of bruises with more than 11 cm when compared with short distance. Most of the bruises found in animals from short distance (49.33%), sized from 1 to 5 cm. For the animals from long distance, the bruises from 1 to 5 cm and higher than 11 cm were the most frequent and the size from 6 to 10 cm was the less frequent. There was independence (*p*-value = 0.6331) in relation to the age of the bruise when associated with the transport distance.

However, there was dependence on the degree of bruise (*p*-value = 0.0124), more evident in degree 1, indicating that the bruises affected only subcutaneous tissue in those transported short distances, those transported over longer distances had bruises in muscle and bone as well.

## 4 Discussion

### 4.1 Behavioral data

The transport of the animals caused stress in the animals, and consequently, due to the hierarchy framework, they tend to show behaviors such as head butting and mounting after unloading.

The behavior of head-butting, which is more common in animals transported over short distances, indicating hierarchy and consequently causing high stress. This behavior was attenuated during lairage time, highlighting the importance of the pre-slaughter rest period. When the animal tries to defend itself against a particular threat or attack, it tends to perform aggressive behaviors, also called agonistic behaviors (29), such as head-butting. This behavior is used by the animal as a way of imposing itself hierarchically (13, 14, 20).

Mounting was more common in animals transported over short distances. This is possibly due to the fact that the cattle are agitated and seek to mark their territory, especially uncastrated males (30). Fighting and TM were observed more frequently in the first few hours after unloading, with a subsequent reduction in the following hours.

Pushing behavior was more observed in animals transported over long distances. Hypothetically, this could be explained by the fact that animals transported over long distances arrived more exhausted at the slaughterhouse, performing behaviors that demand less energy, such as pushing other animals to moving them away, instead of head-butting to promote this action (31).

Cattle transported over short distances preferred to perform the behavior Chasing away compared with those transported over long distances. This is due to the higher level of stress, as the animals have not yet been able to adapt to the transport and/or waiting corral and, therefore, end up performing the behavior more frequently.

The behavioral response to aversive stress is known as the defense response (32) manifested in behaviors related to anxiety, fear, panic, or learned helplessness. Sometimes, this response exhibits a biphasic pattern, which is evident in a *U*-shaped curve. Organism activity decreases when the aversive stimulus is close, increases when it is distant, and decreases again when it is close (32). The proximity of the stimulus is directly correlated with the aversive magnitude of the stimulus. Similarly, transportation stress, which can be observed as a form of containment, can generate biphasic effects, with an initial increase in activity followed by a decrease with continued exposure. The effect is roughly similar to that observed with distance. In other words, a shorter distance elicits a response of lesser magnitude and increases activity, whereas a longer distance exhausts the animal, reducing its overall activity level.

The behaviors NCHBs, MT, PsH, and CA were more practiced in the first hours, with a gradual reduction in the final lairage times, and before slaughter, this may be associated with the animals’ adaptation, indicating improvement in the degree of AW. The greater occurrence of these behaviors in the initial times must be associated with physiological and behavioral changes, due to the stress caused by transport, loading, and unloading, as well as the presence of a new environment, which can affect the final meat quality (33, 34).

### 4.2 Initial and final meat PH

The initial pH of the meat was higher than the final pH, an expected result, as a natural *post mortem* process, when glycogen is converted into lactic acid, as a result of the metabolic process in the absence of O2, accumulating in the muscle tissue, promoting a drop in pH (35). As a consequence, the lower final pH prevents bacterial growth and provides greater tenderness (36–38).

The average final pH value, regardless of the distance traveled, was higher than that indicated for good quality meat, corresponding to 5.5 to 5.8 (39–41), being higher in the carcasses of animals that traveled short distances. This may be associated with the higher number of headbuttings, chasings, and MTs performed by animals transported over short distances due to stress in cattle. In addition, diet, pre-shipment management including unloading steps, transport, and inadequate lairage time can negatively affect carcass pH, interfering with stabilization within the reference parameters of 5.5 to 5.8 (39–41), or because the temperament of zebu cattle is more reactive compared with taurine animals (42–44), especially uncastrated males.

Cattle with a more agitated temperament tend to be more stressed in the pre-slaughter and therefore show less decrease in meat pH (45–49) due to greater consumption of muscle glycogen reserves in the pre-slaughter period.

### 4.3 Initial and final meat RR

RR is considered an excellent short-term stress signal (50). The initial and final RR were above the reference value (10 to 30 respiratory movements per minute – rmp, (21)), indicating that the pre-slaughter management was insufficient to restore parameters compatible with comfort or AW.

High RR values can justify the high final meat pH indicating that the animals were still very agitated at the time of slaughter, which may be a sign that lairage time should be longer.

### 4.4 Bruises on the carcass

Animals transported over short distances had greater damage to the subcutaneous tissue and muscle. This is possibly due to the greater NCHBs practiced in the waiting pen by animals transported over short distances. This information indicates the recommendation to reduce the number of animals inside the waiting pen in order to minimize the factors that lead to conflicts for space between animals that are unloaded when transported over short distances, avoiding agonistic behaviors and physiological changes, which can affect negatively the quality of meat.

Non-castrated male zebu cattle present more agonist and/or aggressive behaviors in relation to taurine animals, have a high response to stressors (51), and consequently release more catecholamines, cortisol, and creatine kinase, which makes it difficult to manage (8, 52–54). Thus, they tend to be more prone to the appearance of bruises (55), especially as a consequence of aggressive behaviors, such as headbutting. The presence of bruises can raise the final pH of meat as consequence of reduction of muscle glycogen and favors the appearance of DFD meat (56–58).

## 5 Conclusion

Lairage time is an effective pre-slaughter tool to decrease undesireble behaviors in resting pen, but transportation distance must be considered, as animals transported over different distance shows different behaviors, indicating that different managements in pre-slaughter pen are required for different transportation distances to ensure meat quality and animal welfare.

The time in the holding pen improved the level of AB (Agonistic Behavior) in all animals, as it reduced agonistic behaviors before slaughter, which is a positive characteristic. However, there was an issue with carcass injuries, more noticeable in animals transported over short distances. Therefore, there is a need to enhance the management of zebu animals transported over short distances to avoid situations of fights causing stress and injuries that can lead to economic losses and affect meat quality. As recommendations, we suggest the following:

1. Another possibility would be to conduct handling before unloading, such as spraying or bathing the animals.
2. Keeping bulls in groups of herd mates and not mixing groups of bulls just before being loaded onto the farm.
3. Provide a larger holding area for these animals to rest, as this may minimize conflicts and improve animal welfare.

## Data availability statement

The original contributions presented in the study are included in the article/supplementary material, further inquiries can be directed to the corresponding author.

## Ethics statement

The animal study was approved by the experimental procedures were carried out in accordance with Brazilian laws and previously approved by the Animal Ethics Committee (CEUA/UFPA), under protocol number 9307300720. The study was conducted in accordance with the local legislation and institutional requirements.

## Author contributions

WS: Formal analysis, Funding acquisition, Investigation, Methodology, Project administration, Resources, Software, Validation, Writing – original draft, Writing – review & editing, Conceptualization. AG: Conceptualization, Investigation, Methodology, Software, Supervision, Validation, Visualization, Writing – original draft, Writing – review & editing. MD: Investigation, Methodology, Resources, Writing – original draft, Writing – review & editing. LS: Conceptualization, Investigation, Methodology, Software, Supervision, Validation, Visualization, Writing – original draft, Writing – review & editing. AB: Formal analysis, Investigation, Methodology, Writing – original draft, Writing – review & editing. ÉB: Investigation, Methodology, Writing – original draft, Writing – review & editing. MS: Investigation, Methodology, Writing – original draft, Writing – review & editing. JL-J: Formal analysis, Investigation, Methodology, Supervision, Writing – original draft, Writing – review & editing. RC: Investigation, Methodology, Writing – original draft, Writing – review & editing. AS: Conceptualization, Formal analysis, Funding acquisition, Investigation, Methodology, Project administration, Resources, Software, Supervision, Validation, Visualization, Writing – original draft, Writing – review & editing.
